# Supplementary material for: Sustainable Valorization of Industrial Cherry Pomace: A Novel Cascade Approach Using Pulsed Electric Fields and Ultrasound Assisted-Extraction
Source: Foods. 2024 Mar 28;13(7):1043. doi: 10.3390/foods13071043 (PMC11012044; doi:10.3390/foods13071043)
Supplement: Supplementary file 1 [file foods-13-01043-s001.zip › foods-2892939-supplementary.pdf]

SUPPLEMENTARY MATERIAL

Sustainable Valorization of Industrial Cherry Pomace: A Novel Cascade Approach Using Pulsed Electric Fields and Ultrasound Assisted-Extraction

1. Cascade of pulsed electric fields (PEF) and ultrasound (US)-assisted extraction experiments

1. Materials and Methods

1.2 Experimental procedure

Figure S1 depicts the comprehensive two-step experimental methodology used for the valorization of cherry pomace, consisting of a sequential process involving Pulsed electric fields (PEF) pre-treatment and the subsequent optimization of ultrasound-assisted extraction (UAE). The detailed equipment setup for both PEF- and UAE-assisted extraction is reported in section 2.2 of the manuscript.

In the first step, 80 g of cherry pomace underwent PEF pre-treatment at optimal conditions (E = 3 kV/cm, W_T = 10 kJ/kg) and then underwent solid-liquid extraction (SLE) using a 50% ethanol-water solvent (1:5 g/mL) at 50 °C for different extraction times (5-60 min).

The residual biomass remaining after PEF-assisted extraction underwent a second step involving UAE to further enhance the recovery yield of bioactive compounds. The sample was transferred to a 1000 mL Erlenmeyer flask and resuspended in a 50% (v/v) ethanol-water mixture at a solid-to-liquid ratio of 1:5 g/mL. To prevent excessive heating during US treatment, the flask was placed in an ice-water bath. The sonication treatments were systematically performed as part of an optimization process aimed at determining the most effective parameters for UAE. These parameters included the treatment power (100, 200, and 400 W) and duration (5-20 min), which played a critical role in maximizing the extraction efficiency and yield of bioactive compounds from industrial cherry pomace. The experiments were carried out with the sample at the initial temperature set at 20 ± 1 °C, while the maximum temperature rise was lower than 5 °C. Following US treatment, the flask was moved to an incubator set at 50 °C, where the diffusion process persisted with continuous shaking at 160 rpm for varying durations (5-60 min). This approach aimed to determine the optimal extraction time necessary to achieve maximal extraction efficiency. Upon completion of the optimal UAE conditions, the same downstream processing steps as for PEF-assisted extraction were conducted to obtain the second extract, separated from the spent residual cherry pomace.

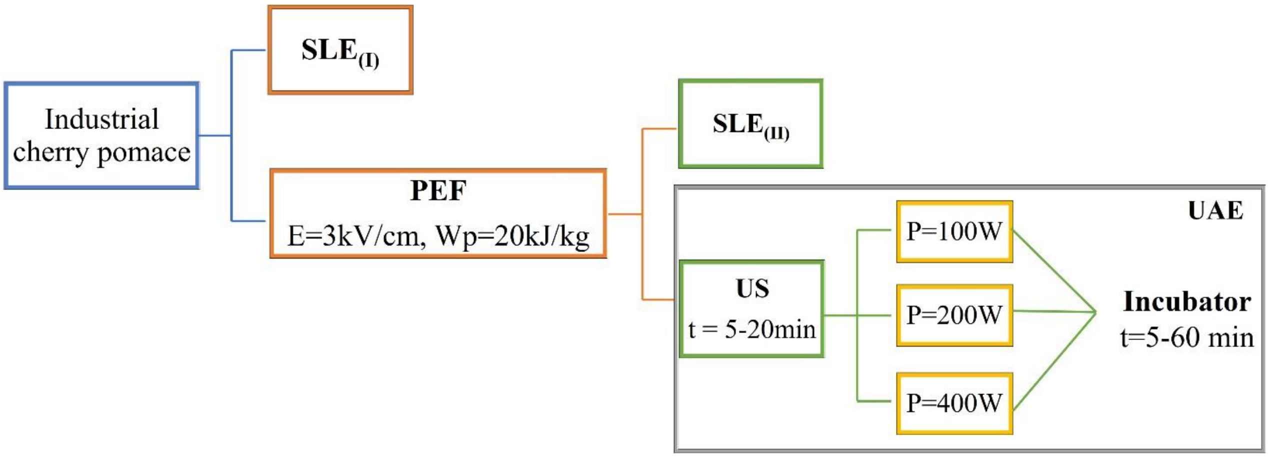

Figure S1. Schematic representation of the cascade PEF-UAE approach.

1.2. Results and discussion

1.2.1. Optimization of UAE process for the recovery of bioactive compounds (TPC, FC, TAC and FRAP).

Figure S2 depicts the results of the second extraction step in terms of total polyphenol content (TPC) (a), flavonoid content (FC) (b), total anthocyanins content (TAC) (c), and antioxidant activity (FRAP) (d) of extracts from untreated ($SLE_{(II)}$) and US-treated (P =100, 200 and 400W, t = 5-20 min, f = 24 kHz, T = 25 °C ± 1 °C) cherry pomace as a function of the extraction time (5+ - 60+ min). The extraction process utilized a 50% (v/v) ethanol-water mixture as the solvent, with parameters set at T = 50 °C, pH = 2.5, and S/L = 1:5 g/mL.

The results delineate a nuanced interplay between sonication power levels and subsequent incubation, shaping the extraction process. Initially, the escalation of sonication power from 100 to 400 W enhances extraction yield, attributed to heightened disruption of cell walls and subsequent release of bioactive compounds. However, during incubation, a notable shift occurs, favouring the 200 W treatment over higher power settings, indicating a delicate balance between effective extraction and preservation of sensitive compounds. This is because larger resonant bubbles, influenced by higher ultrasonic power, result in greater implosion impact, causing tissue fragmentation, pore development, improved mixing, and enhanced diffusivity, ultimately increasing extraction yield [1,2]. This optimal balance likely facilitates deeper solvent penetration into the cellular matrix, enhancing overall extraction efficiency.

Additionally, the progressive enhancement in compound extraction with prolonged sonication duration underscores the importance of extended treatment periods. Beyond this optimal duration, the benefits of additional sonication time become less pronounced. Also, the extended sonication not only promotes particle re-agglomeration due to increased collision frequency or attractive forces between smaller particles but also can significantly elevate the temperature of the system [1,3]. Moreover, the extended incubation period allows for comprehensive diffusion of bioactive compounds, with maximal efficiency achieved after 60 min, particularly notable for TPC, TAC, and antioxidant activity (FRAP). Conversely, FC exhibits optimal extraction efficiency after 45 min of extraction.

Based on these findings, 200 W and 20 minutes were selected as the optimal US power and treatment duration for sonication reflecting the ability to balance effective cell disruption with compound preservation, resulting in superior extraction efficiency after both US treatment and incubation step. As optimal time of the subsequent diffusion step, 60 min were selected as the time which maximized the extraction yield of the investigated bioactive compounds.

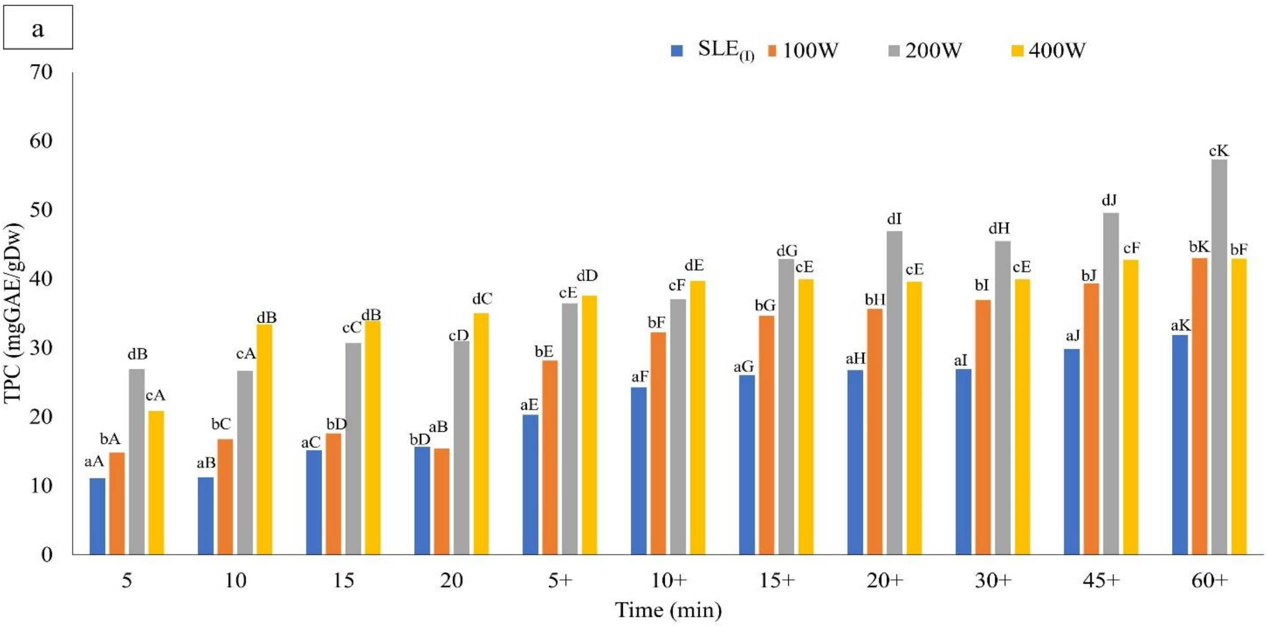
a
SLE(I)
100W
200W
400W
TPC (mgGAE/gDw)
0
10
20
30
40
50
60
70
5
10
15
20
5+
10+
15+
20+
30+
45+
60+
Time (min)

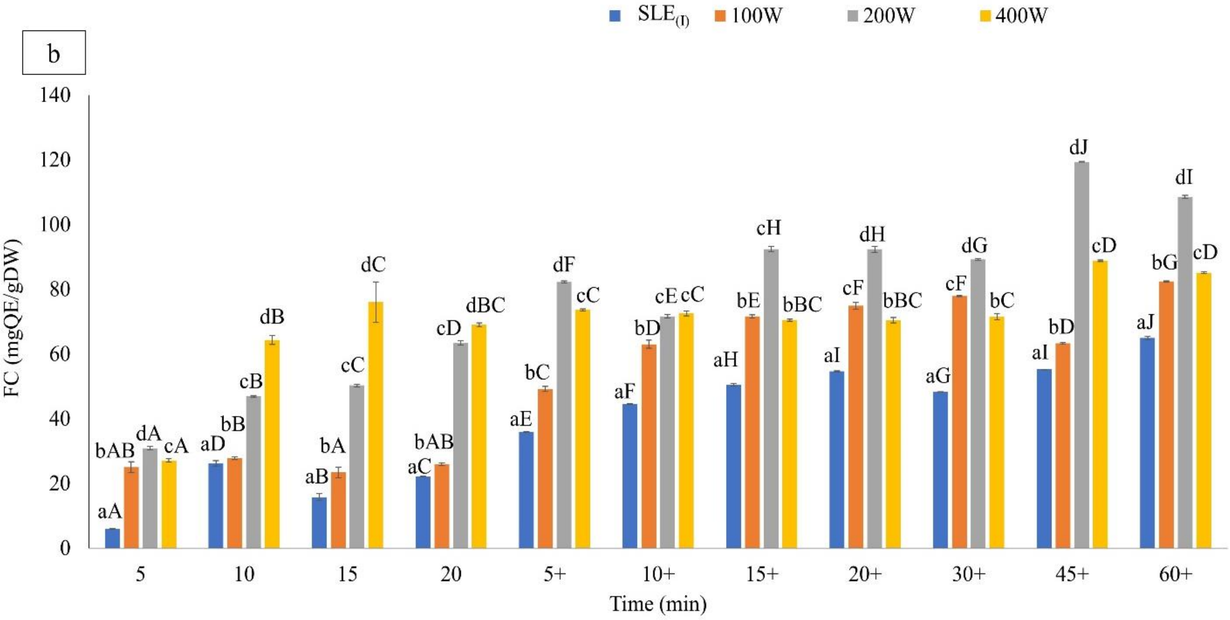
b
SLE(I)
100W
200W
400W
FC (mgQE/gDW)
0
20
40
60
80
100
120
140
5
10
15
20
5+
10+
15+
20+
30+
45+
60+
Time (min)

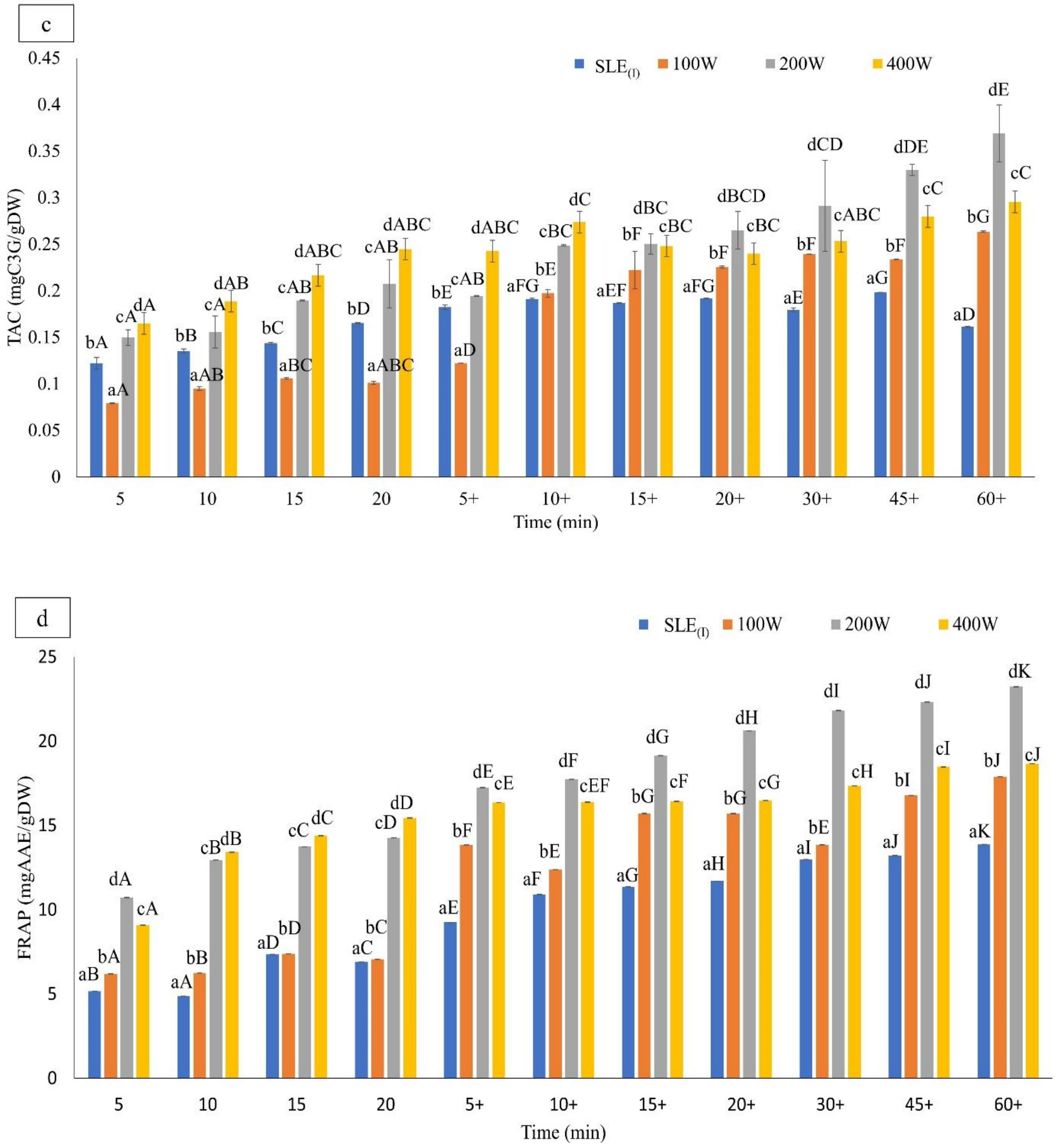

Figure S2. Second extraction step: Total polyphenol content (TPC) (a), flavonoid content (FC) (b), total anthocyanins content (TAC) (c), and antioxidant activity (FRAP) (d) of extracts from untreated ($SLE_{(II)}$) and US-treated (P = 100, 200 and 400 W, t =5- 20 min, f = 24 kHz, T = 25 °C ± 1 °C), cherry pomace as a function of the extraction time (5+ - 60+ min) at EtOH-W 50% (v/v), T = 50°C, t = 60 min, pH = 2.5, and S/L = 1/5 g/mLT of 50 °C, S/L ratio of 1/5 g/mL and pH of 2.5. Data are expressed as mean (n=9) ± standard deviation. Bars with different lowercase letters indicate significant differences ($p \leq 0.05$) between extracts from untreated and US-treated cherry pomace at the same extraction time. Bars with different uppercase letter indicate significant differences ($p \leq 0.05$) among both extracts from untreated and US-treated cherry pomace at different extraction time.

2. References

1. Kumar, K., Srivastav, S., and Sharanagat, V. S. (2021). Ultrasound assisted extraction (UAE) of bioactive compounds from fruit and vegetable processing by-products: A review. *Ultrason Sonochem.* (Vol. 70). Elsevier B.V. doi: 10.1016/j.ultsonch.2020.105325.

2. Maran, J. P., and Priya, B. (2014). Ultrasound-assisted extraction of polysaccharide from *Nephelium lappaceum* L. Fruit peel. International Journal of Biological Macromolecules, 70, 530–536. doi: 10.1016/j.ijbiomac.2014.07.032.

3. González-Centeno, M. R., Knoerzer, K., Sabarez, H., Simal, S., Rosselló, C., and Femenia, A. (2014). Effect of acoustic frequency and power density on the aqueous ultrasonic-assisted extraction of grape pomace (*Vitis vinifera* L.) – A response surface approach. *Ultrason Sonochem*. 21(6), 2176–2184. doi: 10.1016/J.ULTSONCH.2014.01.021.
